# Porous Silicon Oxycarbonitride Ceramics with Palladium and Pd_2_Si Nanoparticles for Dry Reforming of Methane

**DOI:** 10.3390/polym14173470

**Published:** 2022-08-25

**Authors:** Jun Wang, Matthias Grünbacher, Simon Penner, Maged F. Bekheet, Aleksander Gurlo

**Affiliations:** 1 Chair of Advanced Ceramic Materials, Institute of Material Science and Technology, Technische Universität Berlin, Straße des 17. Juni 135, 10623 Berlin, Germany; 2 Institute of Physical Chemistry, University of Innsbruck, Innrain 52c, A-6020 Innsbruck, Austria

**Keywords:** polysilazane, metal-containing precursors, polymer-derived ceramics, palladium nanoparticles, catalyst support, dry reforming of methane

## Abstract

Pd-containing precursor has been synthesized from palladium acetate and poly(vinly)silazane (Durazane 1800) in an ice bath under an argon atmosphere. The results of ATR-FTIR and NMR characterizations reveal the chemical reaction between palladium acetate and vinyl groups in poly(vinyl)silazane and the hydrolyzation reaction between –Si–H and –Si–CH=CH_2_ groups in poly(vinyl)silazane. The palladium nanoparticles are in situ formed in the synthesized precursors as confirmed by XRD, XPS, and TEM. Pd- and Pd_2_Si-containing SiOCN ceramic nanocomposites are obtained by pyrolysis of the synthesized precursors at 700 °C, 900 °C–1100 °C in an argon atmosphere. The pyrolyzed nanocomposites display good catalytic activity towards the dry reforming of methane. The sample pyrolyzed at 700 °C possesses the best catalytic performance, which can be attributed to the in situ formed palladium nanoparticles and high BET surface area of about 233 m^2^ g^−1^.

## 1. Introduction

Silicon-based polymer-derived ceramics (PDCs) have received considerable attention in recent decades due to their low-cost synthesis, simple processing methodology, and good physical and chemical properties, such as high thermal and chemical stability, excellent oxidation and corrosion resistance, as well as out-standing thermomechanical properties [1,2,3]. Moreover, the well-controlled pyrolysis of preceramic precursors at intermediate temperatures under inert atmospheres is decisive in developing micro- and mesoporous components with high surface area [4,5,6,7,8]. Owing to these unique properties, PDCs have been extensively studied for various engineering applications, such as coatings [9], sensors [10], battery anodes [11,12,13], and membranes [14]. In addition, micro-mesoporous PDCs with high surface areas have been reported as promising catalytic supports for several metal catalysts [15,16,17,18]. Embedding metal nanoparticles in PDCs supports could enhance their chemical stability and prevent metal nanoparticles from sintering under operating conditions. For example, metal-containing (e.g., Co and Ni) SiOC nanocomposites have been reported to have good catalytic activity for CO_2_ methanation and Fischer–Tropsch Synthesis [15,17,19,20,21,22]. Metals-containing (e.g., Cu, Ru, Pd, and Ir) SiCN nanocomposites have also been studied as heterogeneous catalysts for liquid-phase oxidation [23], hydrogenation [24,25], and sustainable synthesis [26].

Metal-containing PDCs nanocomposites have usually been synthesized by the thermolysis of precursors composed of preceramic polymers and metal/metal oxide powders under inert atmospheres [27,28,29,30]. However, it is challenging to control the porosity, size, and distributions of the metal nanoparticles in PDCs matrices by applying this synthesis approach. Thus, an alternative approach, based on the in situ formation of homogeneously distributed metal nanoparticles in microporous/mesoporous PDCs matrix during the chemical reaction of organosilicon polymers with metallorganic compounds (e.g., metal carbonyls [31], metal alkoxides [32], metal acetylacetonates [33], and bis(cyclopentadienyl) metal dichlorides (Cp_2_MCl_2_) [34]), has been recently applied. However, some of these metallorganic compounds are extremely harmful and/or need harsh reaction conditions to react with the preceramic polymers. We have recently succeeded in preparing several M/SiOCN nanocomposites (M = Mn, Fe, Co, Cu, Zn, and Ag, Ni, Sn) by chemical reaction of poly(vinyl)silazane (Durazane 1800) with metal acetates followed by pyrolysis of as-derived precursors [13,18,35]. Compared with previous approaches, our synthesis method required very simple reaction conditions (in an ice bath under an argon atmosphere), inexpensive poly(vinyl)silazane (Durazane 1800), and easily accessible commercial metal acetate materials. Moreover, most of the transition metals were in situ formed as spherical nanoparticles in the synthesized precursors during the reaction at room temperature.

The dry reforming of methane (DRM) with carbon dioxide is an interesting method for converting these two carbon-containing greenhouse gases into useful feedstock for further chemical processes [36,37]. In fact, this reaction can generate a syngas with the unity CO/H_2_ ratio, suitable for several industrial processes, such as methanol synthesis, oxo-synthesis and Fischer–Tropsch-synthesis. Many efforts have focused on looking for metal catalysts with high-catalytic performances towards this reaction and high resistance to carbon deposition, thus displaying long-term stability. Excellent catalytic performance can be reached using Ni-based catalysts, such as LaNiO_3_ [38,39], La_2_NiO_4_ [40,41], Sm_1.5_Sr_0.5_NiO_4_ [42], Ni/MnO [43,44] catalyst with long-term stability at 800 °C are reported. However, the successful industrial application of these catalysts has been limited by the requirement of high reaction temperatures to obtain an acceptable level of conversion, causing serious catalyst deactivation problems due to carbon deposition and metal sintering at high temperatures. Thus, suitable catalyst systems for DRM with the aim of reaching high yields of syngas at low necessary reaction temperatures could be promising alternatives. Singha et al. [45] have reported that the Pd–CeO_2_ nanocrystals could initiate the dry reforming of methane reaction at much lower temperatures (i.e., 350 °C) in comparison with Ni-based catalysts. Yue et al. [46] designed Pd/SiO_2_ core–shell nanocatalysts for dry reforming of methane with excellent catalytic performance and good coke-resistant performance. The conversions of CO_2_ and CH_4_ were 89% and 83%, respectively, and no significant decrease was observed for >10 h. Ballesteros-Plata et al. [47] used Pd–Nb catalysts supported by commercial silica in the dry reforming of methane. The effect of the Pd/Nb ratio on the catalytic performance was evaluated. Although Pd supported on γ-Al_2_O_3_ and TiO_2_ showed better catalytic activity at 500 °C comparable to that of Rh/γ-Al_2_O_3_ [48], the catalytic properties of Pd-based catalysts for dry reforming have not been intensively investigated despite the lower cost of Pd as compared, e.g., with Rh.

In this work, a Pd-containing precursor has been synthesized from poly(vinyl)silyzane Durazane 1800 with palladium acetate. This chemical modification approach is advantageous owing to the very simple reaction condition (in an ice bath under an argon atmosphere), the use of rather inexpensive poly(vinyl)silazane (Durazane 1800) and easily accessible commercial metal acetate materials. The palladium nanoparticles are directly in situ generated in the polymer matrix after reaction, and micropores have been maintained in the pyrolyzed ceramic nanocomposites. The obtained porous Pd/SiOCN ceramic nanocomposites are tested as catalysts for DRM for the first time. The chemical structure of synthesized Pd-containing precursor and its polymer-to-ceramic transformation process, phase composition, and porosity development in the pyrolyzed ceramic nanocomposites have also been comprehensively investigated.

## 2. Materials and Methods

### 2.1. The Synthesis of Pd-Containing Precursor

All of the synthesis reactions were carried out according to our recently reported method [18,35] as follows. First, 10 g of Durazane 1800 (dur Xtreme Gmbh, Ulm, Germany) was dissolved in anhydrous THF (50 mL) and then added into a dropping funnel. Then, 4 g of Palladium acetate (≥99.9, Sigma-Aldrich, Burlington, MA, USA) was dispersed in 50 mL of anhydrous THF (≥99.9, Sigma-Aldrich, Burlington, MA, USA) and added into a round flask. The flask and dropping funnel were first assembled in the glovebox, and the reaction vessel was immediately removed from the glovebox and submerged in the ice bath (−6 °C) and connected to Schlenk lines. The synthesis process was continued under stirring and argon flow in the ice bath for 2 h and followed by 24 h at room temperature until the end of gas liberation (Figure S1). Finally, the THF was removed under vacuum for at least 5 h, and the synthesized Pd-containing precursor was collected in the glovebox. The synthesized Pd-containing precursor is denoted as Du1800-Pd.

### 2.2. Pyrolysis of the Synthesized Pd-Containing Precursor

Thermolysis of the synthesized Pd-containing precursor was performed in a tube furnace with a Schlenk tube under flowing argon (approximately 40 mL/min) applying four different pyrolysis temperatures (450 °C, 700 °C, 900 °C, 1100 °C). The precursor was cross-linked first at 250 °C for 2 h, followed by a ceramization step at different temperatures (i.e., 450 °C, 700 °C, 900 °C and 1100 °C) for 3 h before cooling to room temperature.A heating and cooling rate of 50 °C/h and 180 °C /h, respectively, were used. The cross-linked sample was denoted as 250Ar-Du1800-Pd, while the ceramic nanocomposites obtained by pyrolyzing Du1800-Pd precursor at 450, 700, 900, and 1100 °C were labeled as 450Ar-Du1800-Pd, 700Ar-Du1800-Pd, 900Ar-Du1800-Pd, and 1100Ar-Du1800-Pd, respectively. For comparison, the pure Durazane 1800 polymer was also cross-linked and pyrolyzed under the same conditions, and the obtained samples were denoted 250Ar-Du1800, 450Ar-Du1800, 700Ar-Du1800, 900Ar-Du1800, and 1100Ar-Du1800.

### 2.3. Catalytic Tests in the Recirculating Batch Reactor

Briefly, a defined amount of ~50 mg of the respective material with a particle size of 10–50 μm was fixed by quartz wool in a home-built quartz glass reactor tube (volume ~43 mL). The sample was heated in a tube furnace from Linn High Therm with temperature control by a Ni/CrNi thermocouple. An MKS Instruments Baratron pressure transducer measured the gas pressure inside the reactor, while a cold cathode ionization gauge from Balzers ensured the low-pressure control. For the experimental recording of the sample temperature and the respective gas pressure, the National Instruments LabVIEW© software (National Instruments, Austin, TX, USA) was used. Pressures as low as 2 × 10^−7^ mbar in the reactor cell were achieved by a Pfeiffer HiCube pump station. The DRM reaction profile was followed by online quadrupole mass spectrometry Balzers QMA 125; QME125-9 (Balzers, Asslar, Germany). CO_2_, CH_4_, CO, and H_2_ were detected by their molecule ions with the *m*/*z* ratios of 44, 16, 28, and 2, respectively. The fragmentation pattern of CO_2_ was determined in separate experiments by measuring pure CO_2_ at various recipient pressures to correct the CO signal. For the catalytic experiments, we used an initial mixture of 10 mol. % CO_2_ and 10 mol. % CH_4_ balanced with Ar (for background correction due to the constant withdrawal of reaction mixture through the capillary into the mass spectrometer), backfilled to a total pressure of 1000 mbar. The temperature program consisted of a constant heating rate of 15 °C between the isothermal periods of 2 h at 500 °C, 600 °C, 700 °C, and 800 °C.

### 2.4. Attenuated Total Reflectance Fourier Transform Infrared (ATR-FTIR) Spectra

Attenuated Total Reflectance Fourier-Transform Infrared (ATR-FTIR) spectra were collected on the synthesized Pd-containing precursors and pyrolyzed samples in the glovebox in the range from 550 cm^−1^ to 4000 cm^−1^ using Nicolet iS5 (Thermo Fisher Scientific, San Francisco, CA, USA) and Specac Golden Gate (Waltham, MA, USA) with a diamond plate Fourier.

### 2.5. X-ray Photoelectron Spectra (XPS)

X-ray photoelectron spectra (XPS) were collected on ESCALAB 250Xi (Thermo Fisher Scientific, San Francisco, CA, USA). The size of the X-ray spot on the sample was 100 µm. The samples for the X-ray photoelectron measurement were prepared in the glovebox by sprinkling a small amount of sample powders onto the surface of sticky carbon conductive tape stuck on the sample holder. All of the XPS spectra were calibrated using the C1s core line with a binding energy of 284.8 eV.

### 2.6. X-ray Diffraction (XRD)

X-ray diffraction (XRD) was conducted using a Bruker AXS D8 ADVANCE with a Bragg Brentano geometry and a Lynx Eye 1D detector with a CoKα1 radiation wavelength of 0.1789 nm (Bruker, Bremen, Germany); 2θ value ranging from 20 to 90°, with step size and time of 0.01° and 0.6 s, respectively.

### 2.7. Inductively Coupled Plasma Optical Emission Spectroscopy (ICP-OES)

The elemental analysis (for Si and Pd) of the pyrolyzed samples was performed with inductively coupled plasma optical emission spectroscopy (ICP-OES) in a Horiba Scientific ICP Ultima2 (Horiba, Kyoto, Japan). The powder samples were digested in an aqueous suspension with the addition of HNO_3_ and HF at 200 °C for 5 h in an autoclave.

### 2.8. Thermal Gravimetry Analysis (TGA) and Mass Spectrometer (MS)

The polymer to ceramic conversion was investigated in nitrogen with a heating rate of 5 °C min^−1^ with thermal gravimetry analysis (TGA) on STA 409 PC LUXX (Netzsch, Bavaria, Germany) coupled with a mass spectrometer OMNi Star GSD 320 (Pfeiffer Vacuum, Asslar, Germany).

### 2.9. Nitrogen Sorption Measurements

The nitrogen sorption measurements were carried out in a QuadraSorb Station 4 apparatus (Quantachrome, Boynton Beach, FL, USA). Isotherms were recorded at 77 K after degassing under a vacuum at 150 °C for 12 h before the actual measurement. The surface area was calculated using Brunauer−Emmett−Teller (BET) calculations. All of the nitrogen sorption data were analyzed using the Quantachrome/QuadraWin software version 5.05.

### 2.10. Transmission Electron Microscopy (TEM)

The transmission electron microscopy (TEM) images were obtained from FEI Tecnai G2 20 S-TWIN equipped with a LaB6-source at 200 kV acceleration voltage (FEI, Hillsboro, OR, USA). The samples for TEM analysis were prepared in a glovebox by dispersing in anhydrous THF, followed by dropping them onto a copper grid covered with carbon film. The particle size distribution was calculated from TEM images with a Nano measurer software by quantifying 100 particles. TEM was also used for the study of selected area electron diffraction (SAED) patterns.

### 2.11. Solid-State NMR Spectra

Solid-state NMR spectra were recorded with a Bruker Avance 400 MHz spectrometer operating at 100.56 MHz for ^13^C, 79.44 MHz for ^29^Si and 399.88 MHz for ^1^H. ^1^H-^13^C and ^1^H-^29^Si cross-polarization magic angle spinning (CP-MAS) NMR experiments were carried out at a MAS rate of 10 kHz using 4 mm MAS HX double resonance probe. The ^1^H π/2 pulse length was 3.1 µs, and two-pulse phase modulation (TPPM) heteronuclear dipolar decoupling was used during acquisition. The spectra were measured using a contact time of 2.0 ms and a recycle delay of 2 s. All ^13^C and ^29^Si spectra are referenced to external TMS at 0 ppm for ^13^C and for ^29^Si using adamantane and TKS (*tetrakis*(trimethylsilyl)silane) as secondary references, respectively.

## 3. Results

### 3.1. The Chemical Structure of the Synthesized Pd-Containing Precursor

**Figure 1:**
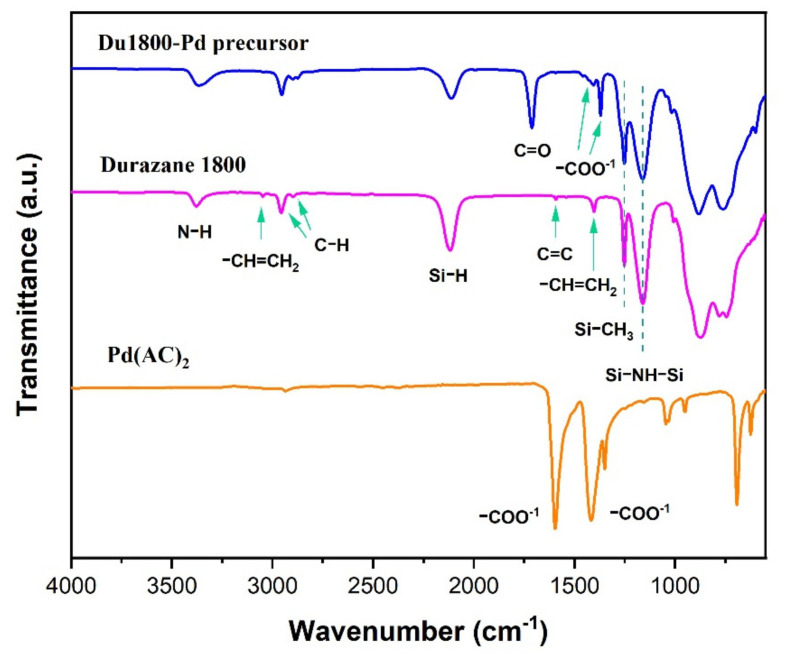
The ATR-FTIR spectra of poly(vinyl)silazane (Durazane 1800) and synthesized Du1800-Pd precursor.

The ATR-FTIR spectra of poly(vinyl)silazane (Durazane 1800) and synthesized Pd-containing (Du1800-Pd) precursor are shown in Figure 1, the spectrum of Durazane 1800 shows the absorption bands of poly(vinyl)silazane: the bands associated with –Si–NH–Si– groups involve N–H stretching at 3382 cm^−1^ and Si–N vibration 1162 cm^−1^. The bands related to the vinylsilyl groups (CH_2_=CH–Si–) include C–H vibrations at 3042 cm^−1^ and 3006 cm^−1^, C=C stretching at 1590 cm^−1^, and the scissoring of terminal methylene at 1402 cm^−1^. The strong absorption band observed at 2116 cm^−1^ can be assigned to Si–H vibration. The characteristic band of the Si–CH_3_ groups is observed at 1255 cm^−1^, while the bands corresponding to the C–H stretching are located at 2953 cm^−1^ and 2896 cm^−1^. Moreover, for the synthesized Du1800-Pd precursor, the C–H vibrations bands at 3042 cm^−1^ and 3006 cm^−1^, which can be assigned to the vinyl groups, vanish, and new bands related to C=O (1712 cm^−1^), acetate anion (1561 cm^−1^ and 1414 cm^−1^) appear [49,50,51]. These results suggest the successful chemical reactions between palladium acetate with poly(vinyl)silazane polymer through the vinyl groups, which agrees with our previous works [18,35].

**Figure 2:**
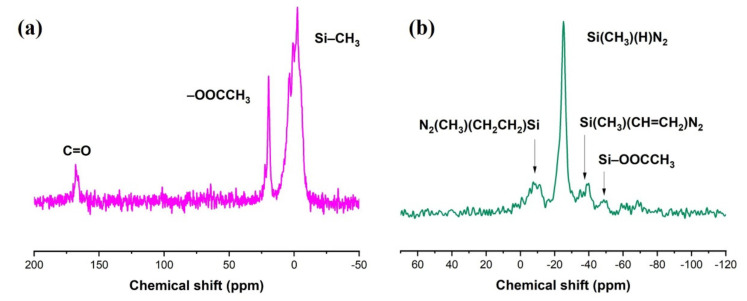
The (**a**) ^13^C NMR spectra and (**b**) ^29^Si NMR spectra of Du1800-Pd precursor.

The chemical structure of the Du1800-Pd precursor was characterized by ^13^C {^1^H} and ^29^Si {^1^H} CPMAS NMR. The ^13^C {^1^H} CPMAS NMR spectrum of Du1800-Pd precursor (Figure 2a) exhibits signals between −10 and 5 ppm, which can be assigned to Si–CH_3_ group. The signals at 20 ppm and 167 ppm, which are not observed in the ^13^C NMR spectrum of Durazane 1800, correspond to the –CH_3_ and C=O groups in acetate anion, respectively. Moreover, no signals attributed to the vinyl group (–Si–CH=CH_2_) between 128 and 142 ppm are observed in the spectrum of Du800-Pd precursor. The ^29^Si {^1^H} CPMAS NMR spectrum (Figure 2b) shows the presence of Si(CH_3_)(CH_2_CH_2_)N_2_ units in the synthesized Du1800-Pd precursor at −8 ppm. The signal observed at −25 and −38 ppm can be assigned to the residue Si(CH_3_)(H)N_2_ and Si(CH_3_)(CH=CH_2_)N_2_ units in the Du1800-Pd precursor, respectively. The weak signals between −40 and −60 ppm might be attributed to Si–OOCCH_3_ units. The ^13^C {^1^H} and ^29^Si {^1^H} CPMAS NMR results confirm the chemical reaction of palladium acetate with polymer Durazane 1800, as well as the hydrosilylation reaction between Si–H and –Si–CH=CH_2_, which agrees with ATR-FTIR results (Figure 1). The FTIR and NMR results suggest the reaction between the acetate groups in palladium acetate with the silicon centers of Durazane 1800 polymer, causing the decrease in the number of Si–H and Si–NH–Si groups in the obtained precursors and the formation of acetosilyl groups (CH_3_COO–Si), palladium nanoparticles. These chemical reactions between palladium acetate and the polymer increase the degree of polymer cross-linking and ceramic yield, as discussed below.

**Figure 3:**
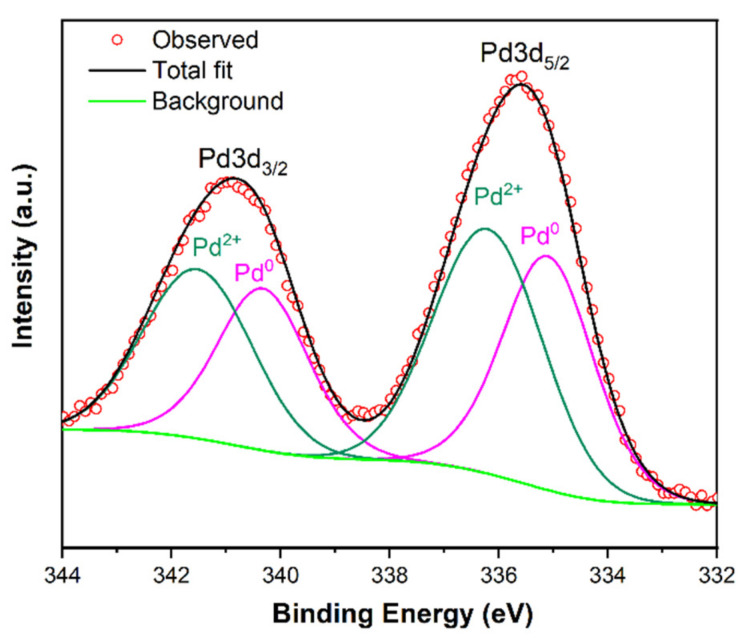
The Pd 3d X-ray photoelectron spectra of the Du1800-Pd precursor.

**Figure 4:**
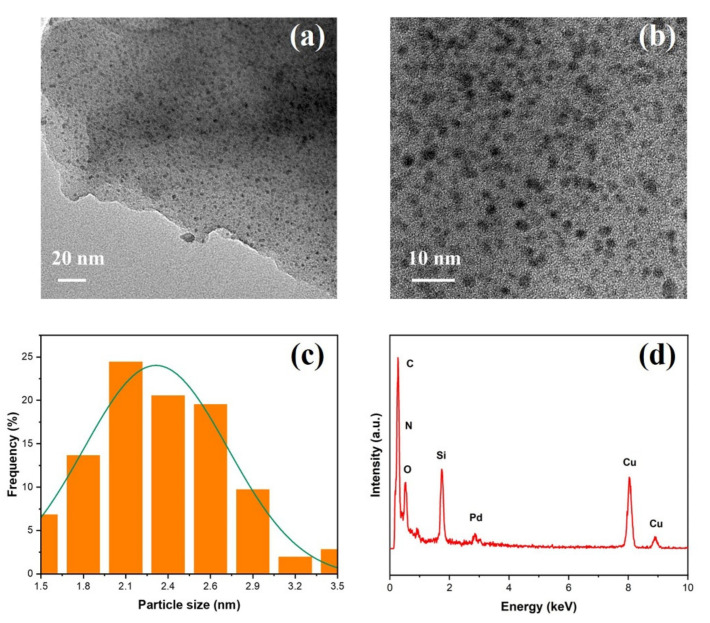
(**a**,**b**) TEM images, (**c**) size distribution of the in situ formed palladium nanoparticles, and (**d**) EDX spectrum of the Du1800-Pd precursor. Cu signals come from the copper grid.

The oxidation states of incorporated palladium on the surface of synthesized Du1800-Pd precursor were investigated by X-ray photoelectron spectroscopy (XPS). As shown in Figure 3, XPS Pd 3d spectra of the Du1800-Pd precursor can be deconvoluted into two doublet peaks characteristic for 3d_3/2_ and 3d_5/2_ spin-orbit splitting. The doublet peak observed at 335.2 eV and 340.4 eV can be assigned to metallic Pd^0^, while the doublet peak at 336.2 and 341.5 eV can be attributed to Pd^2+^, which is in good agreement with the previous reports for metallic Pd and PdO compounds [52]. The ratio of Pd^0^/Pd^2+^ calculated from the area of the peaks is around 0.88. This result suggests metallic palladium phase is in situ formed during the chemical reaction of palladium acetate and polymer Durazane 1800, which is also confirmed by the XRD results (Figure 10). The XRD pattern of the Du1800-Pd precursor manifests clear broad reflection at a 2θ value of 46.7°, which corresponds to the (111) plane of the metallic palladium phase (PDF No. 87-0637). However, the broadening of the XRD reflections suggests the small crystallite size of metallic palladium, which is also consistent with TEM results (Figure 4 and Figure S2). The formation of metallic palladium is also confirmed with the measured interplanar spacing of 0.227 ± 0.02 nm in the SAED pattern (Figure S2), corresponding to the (111) plane. Transmission electron microscopy (TEM) images reveal that the formed palladium nanoparticles are homogeneously distributed in the synthesized Du1800-Pd precursor (Figure 4a,b), and the average particle size of palladium is ~2.3 nm (Figure 4c). EDX analysis shows the presence of the C, N, O, Si, and Pd elements in the amorphous polymer matrix (Figure 4d).

### 3.2. The Transformation of the Synthesized Du1800-Pd Precursor into Ceramic Nanocomposites

**Figure 5:**
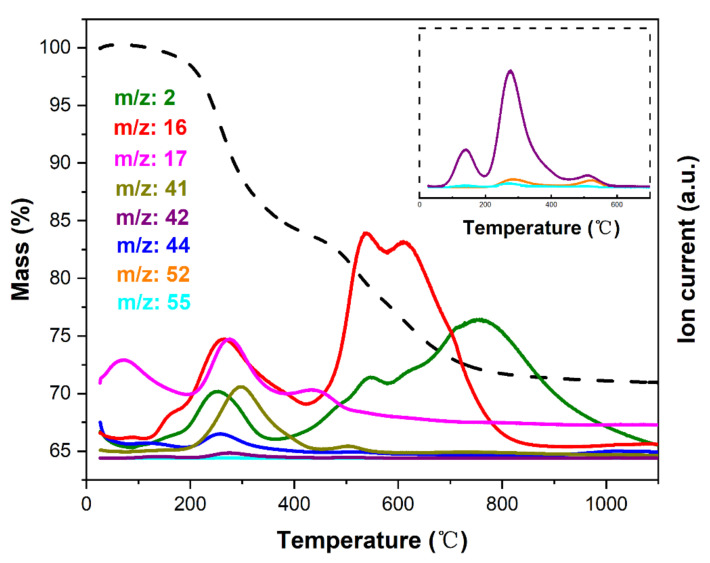
Results of simultaneous gravimetry (dashed lines)-mass spectrometry analysis of the synthesized Du1800-Pd precursor. The inset shows the mass spectra on an enlarged scale.

The polymer-to-ceramic transformation process of Du1800-Pd precursor is investigated by the simultaneous thermal analysis; the results of the STA are displayed in Figure 5. The first weight loss stage (~16%) below 400 °C is accompanied by the release of H_2_ (*m*/*z* = 2), CH_4_ (*m*/*z* = 16), NH_3_ (*m*/*z* = 17), and CO_2_ (*m*/*z* = 44). The CO_2_ evolution at 200–400 °C should be attributed to the decomposition of side groups (i.e., Si–OOCCH_3_ units) in the Du1800-Pd precursor, which has been confirmed by detecting CHCO^+^ (*m*/*z* = 41) and CH_2_CO^+^ (*m*/*z* = 42) ions in the same temperature range. In addition, hydrocarbon oligomers (*m*/*z* = 52 and 55) have also been detected at the temperature range of 200–400 °C, which suggests that the formed metallic palladium nanoparticles in the Du1800-Pd precursor might catalyze the formation of these hydrocarbons. The release of H_2_ and CH_4_ causes the second weight loss stage (~13%) at 400–1100 °C. The Du1800-Pd precursor exhibits a ceramic yield of ~71% after pyrolysis at 1100 °C under an argon atmosphere, which is higher than the ceramic yield of pure Durazane 1800 (~66%).

**Figure 6:**
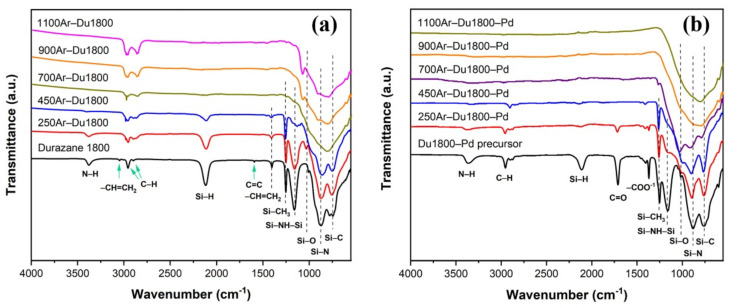
ATR-FTIR spectra of (**a**) Durazane 1800 and (**b**) Du1800-Pd precursor before pyrolysis, after cross-linking at 250 °C for 2 h, followed by ceramization at different temperatures (700 °C, 900 °C and 1100 °C) under argon atmosphere for 3 h.

Ex situ ATR-FTIR was further performed to obtain more information on polymer-to-ceramic transformation processes. As analyzed from Figure 6a, the absorption bands corresponding to vinyl groups (3042 cm^−1^, 3006 cm^−1^, and 1590 cm^−1^) disappear when polymer Durazane 1800 is cross-linked at 250 °C, which indicates cross-linking reactions based on a hydrolyzation reaction between –Si–CH=CH_2_ and Si–H happen at this temperature. A further increase in the pyrolysis temperature to 700 °C results in the occurrence of broad absorption bands corresponding to the vibrations of Si–O (1012 cm^−1^), Si–N (894 cm^−1^), and Si–C (752 cm^−1^), which suggests that the polymer-to-ceramic transformation process is complete. In contrast, for Du1800-Pd precursor (Figure 6b), the intensity of absorption bands corresponding to N–H (3343 cm^−1^), C–H (2960 cm^−1^), Si–H (2152 cm^−1^), C=O (1712 cm^−1^), and –COO^−1^ (1610–1330 cm^−1^) gradually decreases with increasing pyrolysis temperature. The broad absorption bands due to Si–O (1026 cm^−1^), Si–N (904 cm^−1^), and Si–C (766 cm^−1^) can be observed when the precursor is pyrolyzed at 900 and 1100 °C.

### 3.3. The Chemical Composition and Microstructure of the Pyrolyzed Ceramic Nanocomposites

**Figure 7:**
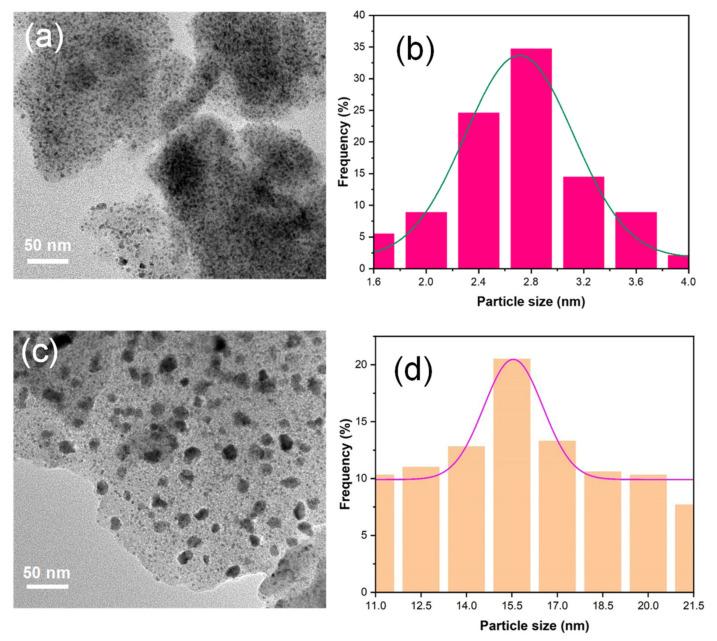
TEM image of (**a**) 700Ar-Du1800-Pd and (**c**) 1100Ar-Du1800-Pd samples. (**b**,**d**) Particle size distribution of Pd and Pd_2_Si nanoparticles in the 700Ar-Du1800-Pd and 1100Ar-Du1800-Pd samples, respectively.

The chemical compositions of obtained Pd/SiOCN ceramic nanocomposites were characterized using ICP-OES, XRD, and TEM. As shown in Table S1, although the weight content of Pd and Si in the samples increases with pyrolysis temperatures, the ratio of Si/Pd in all of the samples is about 2.1 ± 0.1. These results are expected as the light elements in the precursor, such as C, O, H, and N will be lost as gases with increasing pyrolysis temperatures. Based on the ceramic yield of pure Durazane 1800 (~66%) at 1100 °C and the theoretical value of weight loss of pure palladium acetate to metallic palladium (52.6%), the palladium content is estimated to be 22% in the 1100Ar-Du1800-Pd sample. As shown in Table S1, this value is slightly higher than that determined by ICP analysis, which can be explained by the increase in the ceramic yield by reacting palladium acetate with the polymer (Figure 5). The partial loss of acetate groups in palladium acetate due to the reaction with the polymer could be another reason. As shown in Figure 10, the XRD pattern of 700Ar-Du1800-Pd sample reveals the presence of nanocrystalline metallic palladium (PDF No. 87-0637) and palladium silicide Pd_2_Si (PDF No. 89-3048). The metallic palladium phase further reacts with SiOCN matrix at higher pyrolysis temperatures to form palladium silicide, as revealed from the XRD pattern of the 900Ar-Du1800-Pd and 1100Ar-Du1800-Pd samples obtained by the pyrolysis of the precursor at 900 °C and 1100 °C, respectively. TEM analysis of the 700Ar-Du1800-Pd (Figure 7a and Figure S2b) and 1100Ar-Du1800-Pd (Figure 7c) samples confirms the predominantly amorphous nature of the SiOCN matrix and the formation of fine nanoparticles with an average particle size of ~2.7 nm and 15.5 nm, respectively, that are homogeneously distributed in an amorphous SiOCN ceramic matrix. These results suggest that the average particle size of the formed Pd and Pd_2_Si increase with the pyrolysis temperature due to the sintering of the particles at high temperatures. The SAED pattern (inset of Figure S2b) confirms the presence of metallic palladium as indicated from the diffraction ring assigned to the (111) lattice plane of its face-centered cubic (fcc) structure. These results are corroborated with the XRD results (Figure 10).

**Figure 8:**
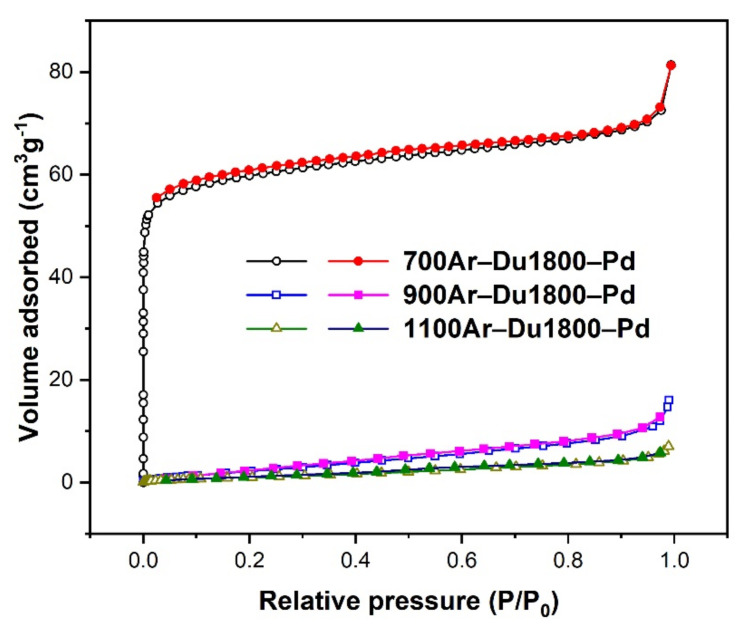
Nitrogen physisorption isotherms of the samples derived from Du1800-Pd precursor (open and filled symbols indicate the adsorption and desorption stage, respectively).

Figure 8 shows the nitrogen physisorption isotherms of samples derived from Du1800-Pd precursor at different pyrolysis temperatures. The 700Ar-Du1800-Pd sample exhibits type I isotherm corresponding to microporous materials and BET specific surface area of ~233 m^2^ g^−1^. In contrast, the 900Ar-Du1800-Pd and 1100Ar-Du1800-Pd samples are nonporous with low BET specific surface areas of ~11 and ~5 m^2^ g^−1^, respectively.

### 3.4. The Catalytic Properties of Carbon Dioxide Dry Reforming of Methane

**Figure 9:**
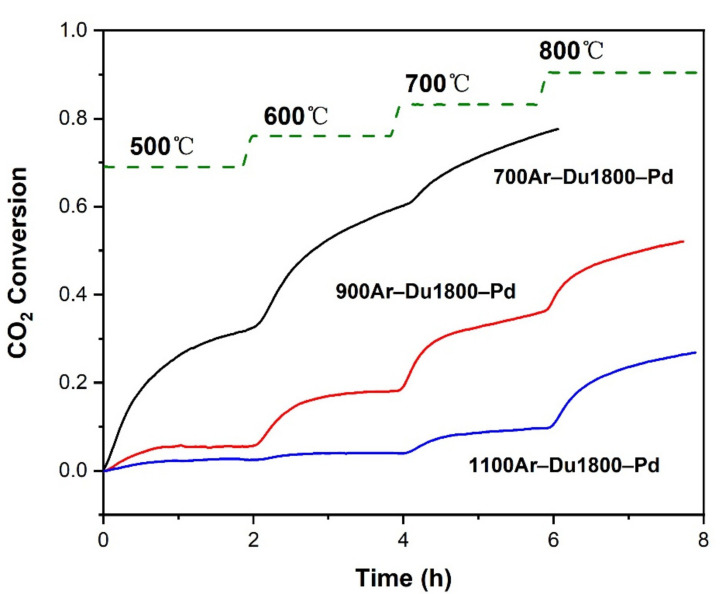
CO_2_ conversion in the DRM tests in a recirculating batch reactor with an initial CH_4_:CO_2_:Ar= 1:1:8 mixture at 1000 mbar initial pressure.

The catalytic performance of the obtained Pd/SiOCN ceramic nanocomposites derived from Du1800-Pd precursor are studied in a recirculating batch reactor initially containing CH_4_:CO_2_:Ar = 1:1:8 mixtures at 1000 mbar of pressure. The CO_2_ conversion with time in the temperature range of 500–800 °C is shown in Figure 9. All of the samples display catalytic activity towards DRM, as confirmed by the conversion of CO_2_ and the detection of CO and H_2_ in the MS spectra (Figure S3). The CO_2_ conversion of all samples increases with increasing temperature due to the endothermic nature of the DRM process. The 700Ar-Du1800-Pd sample shows the best catalytic performance with a CO_2_ conversion of 78% at 700 °C, which is almost the same performance reported for Ni/SiOCN catalysts measured in the same catalytic reactor and under the same conditions [18], as shown in Figure S4.

To explain the catalytic performance, post-catalytic characterizations are applied to the spent catalysts. The Pd 3d XPS spectra of all of the samples before and after DRM are shown in Figure S5a. The doublet at 341.2/336.3 eV that is associated with Pd^2+^ disappears after the DRM test, which suggests the reduction of the PdO phase in the samples to metallic palladium or palladium silicide under reducing DRM conditions. As shown in Figure S5b, the C 1s XPS spectra of all samples before and after DRM can be fitted with four peaks at 283.7, 284.7, 286.0 and 288.3 eV, which can be assigned to C–Si, C–C, C–N and C-O bonds, respectively. Moreover, the analysis of the XPS survey (Table S2) reveals that the amount of surface carbon in the spent 700Ar-Du1800-Ni catalyst after DRM is the highest among all samples.

**Figure 10:**
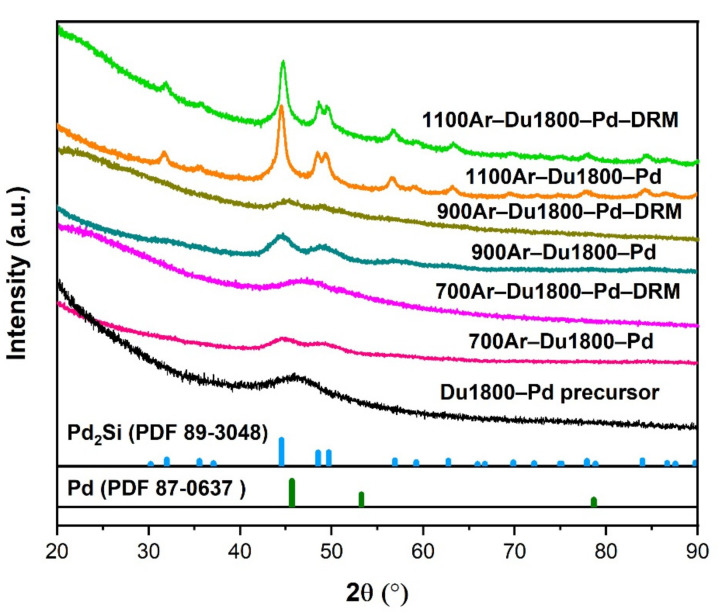
XRD patterns of the Du1800-Pd precursor and their derived Pd/SiOCN ceramic nanocomposites before and after the DRM tests in a recirculating batch reactor.

The XRD patterns of the catalysts before and after DRM are shown in Figure 10. For the 700Ar-Du1800-Pd and 900Ar-Du1800-Pd catalysts, the XRD reflections corresponding to the Pd and Pd_2_Si phase become broader after DRM, suggesting the decrease in the crystallinity of the sample due to the transformation of metallic Pd into Pd_2_Si phase. In contrast, the XRD pattern of 1100Ar-Du1800-Pd catalyst generally reveals no noteworthy phase transitions during the DRM process.

**Figure 11:**
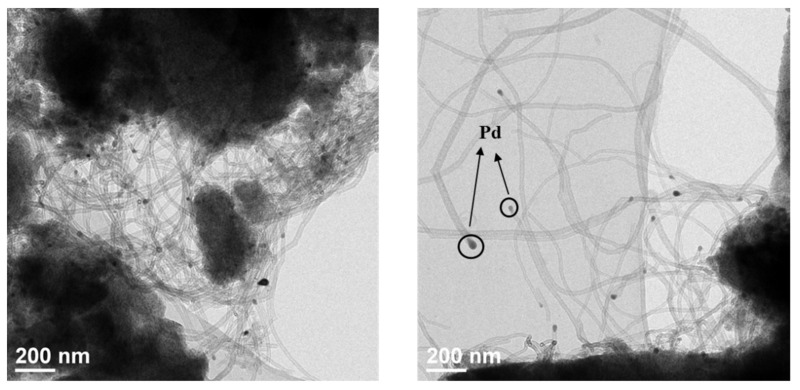
The TEM images of the spent 700Ar-Du1800-Pd catalyst after the DRM tests in a recirculating batch reactor.

Figure 11 shows the TEM images collected on the spent 700Ar-Du1800-Pd catalyst after the DRM tests. A high amount of multiwalled sp^2^-carbon nanotubes is formed on the surface of the spent catalyst.

## 4. Discussion

In the present study, all the palladium-containing samples show activity toward syngas formation, and substantial differences appear between them. The 700Ar-Du1800-Pd sample displays the highest conversion all along with the temperature range, achieving the highest final conversion ~0.78 after a 6 h reaction. The reason for the characteristic performance of these samples can be explained by differences in the catalyst configuration, which includes the surface area (Figure 8) and chemical compositions (Figure 10). The slope of CO_2_ conversion decreases with time at a given temperature, which can be explained by the following possible mechanisms: (i) the decreased reactants’ partial pressure in the batch reactor due to the continuous depletion of CH_4_ and CO_2_. (ii) The sintering of active palladium particle sintering (the decrease in active surface area). (iii) The transformation of active metallic palladium into its silicide phase displays a diminished activity toward syngas formation [53,54].

Surface carbon resulting from CO disproportionation or CH_4_ decomposition has been stated to deactivate the catalysts or also to be involved in the reaction. The presence of surface carbons can act as an intermediate toward the generation of syngas by oxidation with CO_2_ under specific conditions. Thus, they are not necessarily linked to catalyst deactivation. In fact, the high increase in the content of surface carbon (Table S2) for the best-performing samples suggests that surface carbon is involved in the reaction mechanism. However, the formation of surface carbon cannot be confirmed by XRD (surface carbon can be amorphous or its low amount is below the technique’s detection limit).

## 5. Conclusions

Pd-containing polysilazane precursor has been synthesized via the chemical reaction of Pd(Ac)_2_ and poly(vinyl)silazane Durazane 1800. Palladium nanoparticles of about 2–3 nm in diameter are in situ formed in the synthesized precursor; they partially react with a silicon-containing matrix to form palladium silicide Pd_2_Si during the pyrolysis under an argon atmosphere. The 700Ar-Du1800-Pd ceramic nanocomposites are found to be microporous materials with a high BET specific surface area of ~233 m^2^ g^−1^, while both 900Ar-Du1800-Pd and 1100Ar-Du1800-Pd samples are nonporous materials with low BET specific surface area of ~11 and ~5 m^2^ g^−1^, respectively. The catalytic tests for the dry reforming of the methane process reveal that all of the samples are active, but the 700Ar-Du1800-Pd sample shows the best performance.

## Data Availability

Not applicable.

## Supplementary Materials

The following supporting information can be downloaded at: https://www.mdpi.com/article/10.3390/polym14173470/s1, Figure S1: Colour changes with time during the chemical reaction of palladium acetate with poly(vinyl)silazane Durazane 1800.; Figure S2: HRTEM image of (a) the Du1800-Pd precursor and (b) 700Ar-Du1800-Pd samples. The corresponding SAED patterns are shown in the insets., Figure S3: The normalized CO_2_, CO, and H_2_ MS signal in the DRM test in a recirculating batch reactor for (a) 700Ar-Du1800-Pd (b) 900Ar-Du1800-Pd (c) 1100Ar-Du1800-Pd samples., Figure S4: CO_2_ conversion of the Ni/SiOCN catalysts (900Ar-Du1800-Ni sample in reference [18]) in the DRM tests in a recirculating batch reactor with an initial CH_4_:CO_2_:Ar= 1:1:8 mixture at 1000 mbar initial pressure., Figure S5: The Pd 3d and C 1s XPS spectra of samples before and after DRM in a recirculating batch reactor., Table S1: The Si and Pd contents in the pyrolyzed samples (ICP-OES results) and their BET surface area., Table S2: Element compositions of the samples before and after DRM (X-ray photoelectron spectroscopy).
