# Supplementary material for: Porous Silicon Oxycarbonitride Ceramics with Palladium and Pd2Si Nanoparticles for Dry Reforming of Methane
Source: Polymers (Basel). 2022 Aug 25;14(17):3470. doi: 10.3390/polym14173470 (PMC9460865; doi:10.3390/polym14173470)
Supplement: Supplementary file 1 [file polymers-14-03470-s001.zip › polymers-1779341-supplementary.pdf]

polymers

MDPI

Supporting Information

Porous Silicon Oxycarbonitride Ceramics with Palladium and Pd_2Si Nanoparticles for Dry Reforming of Methane

Jun Wang [1], Matthias Grünbacher [2], Simon Penner [2], Maged F. Bekheet [1,*] and Aleksander Gurlo [1]

1 Chair of Advanced Ceramic Materials, Institute of Material Science and Technology, Technische Universität Berlin, Straße des 17. Juni 135, 10623 Berlin, Germany
2 Institute of Physical Chemistry, University of Innsbruck, Innrain 52c, A-6020 Innsbruck, Austria
* Correspondence: maged.bekheet@ceramics.tu-berlin.de

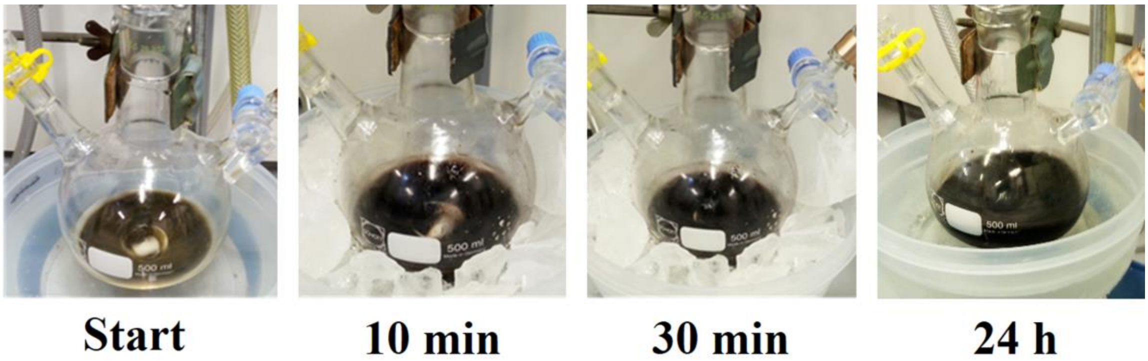

Figure S1. Colour changes with time during the chemical reaction of palladium acetate with poly(vinyl)silazane Durazane 1800.

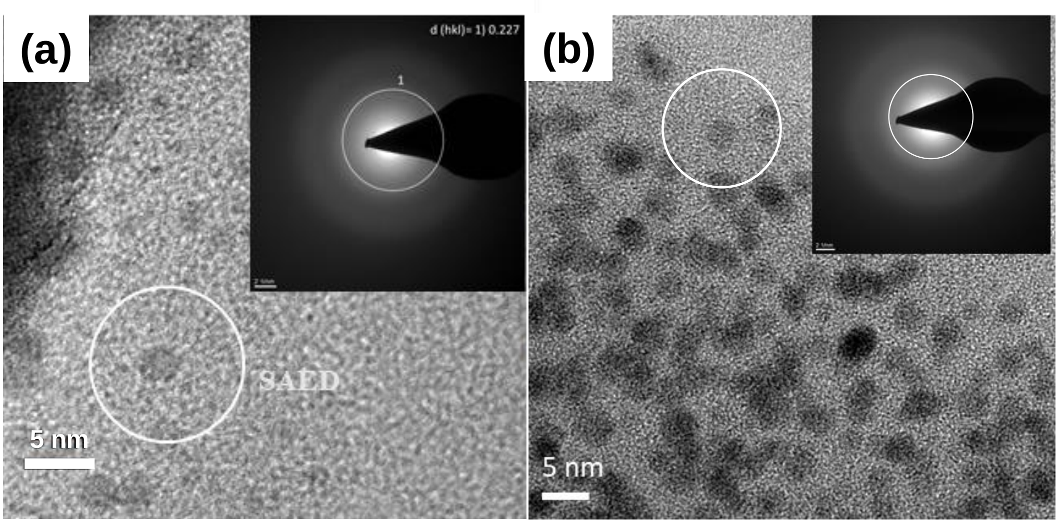

Figure S2. HRTEM image of (**a**) the Du1800-Pd precursor and (**b**) 700Ar-Du1800-Pd samples. The corresponding SAED patterns are shown in the insets.

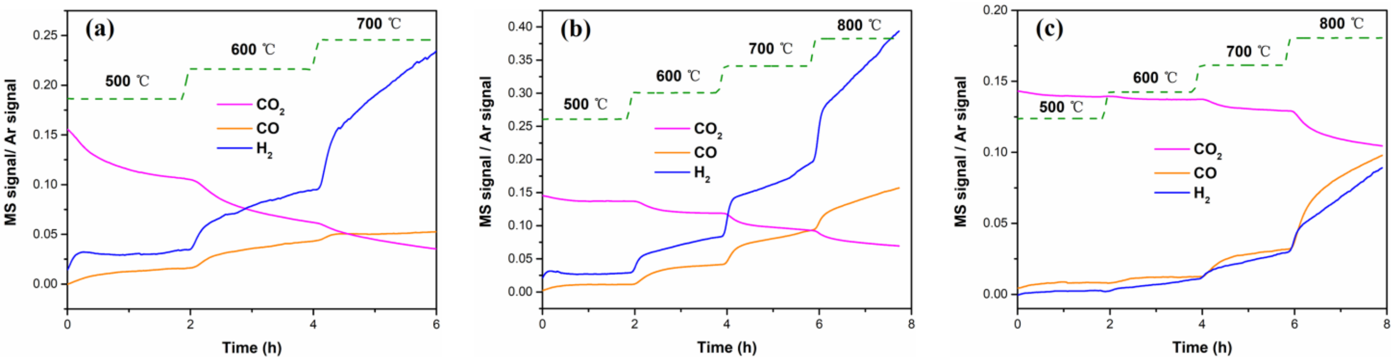

Figure S3. The normalized CO_2, CO, and H_2 MS signal in the DRM test in a recirculating batch reactor for (**a**) 700Ar-Du1800-Pd (**b**) 900Ar-Du1800-Pd (**c**) 1100Ar-Du1800-Pd samples.

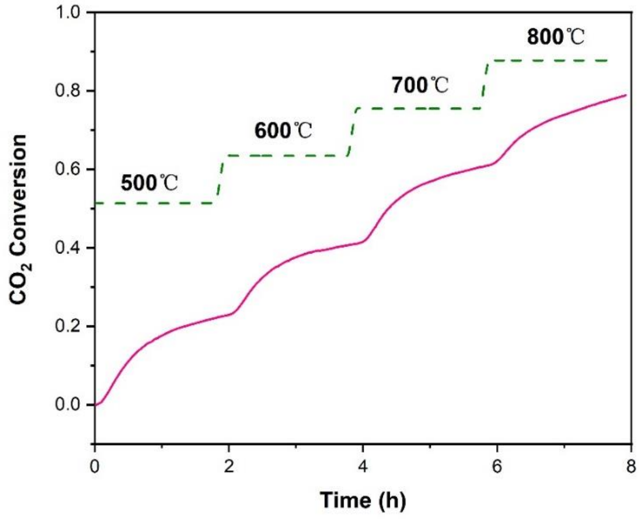

Figure S4. CO_2 conversion of the Ni/SiOCN catalysts (900Ar-Du1800-Ni sample in reference [18]) in the DRM tests in a recirculating batch reactor with an initial CH_4:CO_2:Ar= 1:1:8 mixture at 1000 mbar initial pressure.

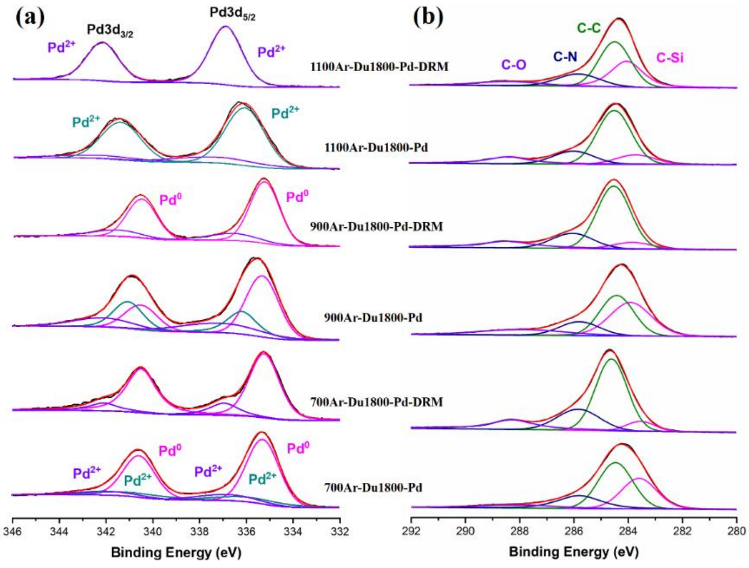

Figure S5. The Pd 3d and C 1s XPS spectra of samples before and after DRM in a recirculating batch reactor.

Table S1. The Si and Pd contents in the pyrolyzed samples (ICP-OES results) and their BET surface area.

Sample	Si content (wt %)	Pd content (wt %)	Si/Pd (weight ratio)		BET surface area (m2 g-1)
			Expected	Observed	
700Ar-Du1800-Pd	29.6	13.5		2.2	232.7
900Ar-Du1800-Pd	32.0	15.4		2.1	11.2
1100Ar-Du1800-Pd	37.2	17.3	1.6	2.2	4.6

Table S2. Element compositions of the samples before and after DRM (X-ray photoelectron spectroscopy).

Sample	Si (at%)	C (at%)	N (at%)	O (at%)	Pd (at%)	Atomic ratio			
						C/Si	N/Si	O/Si	Pd/Si
700Ar-Du1800-Pd	28.18	22.48	11.59	36.45	1.31	0.80	0.41	1.29	0.05
700Ar-Du1800-Pd -DRM	14.04	61.32	5.31	18.76	0.58	4.37	0.38	1.34	0.04
900Ar-Du1800-Pd	25.32	24.06	11.57	37.62	1.43	0.95	0.46	1.49	0.05
900Ar-Du1800-Pd-DRM	24.01	31.85	4.83	38.46	0.64	1.33	0.20	1.60	0.03
1100Ar-Du1800-Pd	22.02	37.53	11.04	28.34	1.06	1.70	0.50	1.29	0.05
1100Ar-Du1800-Pd -DRM	27.83	26.91	9.11	35.33	0.60	0.97	0.33	1.27	0.02
